# Activation of Secondary Metabolite Gene Clusters in *Streptomyces clavuligerus* by the PimM Regulator of *Streptomyces natalensis*

**DOI:** 10.3389/fmicb.2019.00580

**Published:** 2019-03-26

**Authors:** Yolanda Martínez-Burgo, Javier Santos-Aberturas, Antonio Rodríguez-García, Eva G. Barreales, José Rubén Tormo, Andrew W. Truman, Fernando Reyes, Jesús F. Aparicio, Paloma Liras

**Affiliations:** ^1^Microbiology Section, Department of Molecular Biology, University of León, León, Spain; ^2^Department of Molecular Microbiology, John Innes Centre, Norwich, United Kingdom; ^3^Institute of Biotechnology of León, INBIOTEC, León, Spain; ^4^Centre of Excellence for Research into Innovative Medicine, Health Sciences Technology, MEDINA, Granada, Spain

**Keywords:** *Streptomyces clavuligerus*, PimM, PAS-LuxR regulator, transcriptomics, secondary metabolite

## Abstract

Expression of non-native transcriptional activators may be a powerful general method to activate secondary metabolites biosynthetic pathways. PAS-LuxR regulators, whose archetype is PimM, activate the biosynthesis of polyene macrolide antifungals and other antibiotics, and have been shown to be functionally preserved across multiple *Streptomyces* strains. In this work we show that constitutive expression of *pimM* in *Streptomyces clavuligerus* ATCC 27064 significantly affected its transcriptome and modifies secondary metabolism. Almost all genes in three secondary metabolite clusters were overexpressed, including the clusters responsible for the biosynthesis of the clinically important clavulanic acid and cephamycin C. In comparison to a control strain, this resulted in 10- and 7-fold higher production levels of these metabolites, respectively. Metabolomic and bioactivity studies of *S. clavuligerus::pimM* also revealed deep metabolic changes. Antifungal activity absent in the control strain was detected in *S. clavuligerus::pimM*, and determined to be the result of a fivefold increase in the production of the tunicamycin complex.

## Introduction

PimM is a PAS-LuxR type regulator that is critical for the regulation of the biosynthetic gene cluster of pimaricin, a polyene macrolide antifungal compound produced by *Streptomyces natalensis* (Antón et al., 2007; Barreales et al., 2018). This protein carries a PAS-sensor motif at its N-terminus and a helix-turn-helix (HTH) motif at the C-terminus. Expression of *pimM* is controlled by a second regulator, PimR (Santos-Aberturas et al., 2012), and disruption of *pimM* results in a mutant unable to produce pimaricin (Antón et al., 2007). Homologous regulators are encoded by genes located in the clusters for other polyene macrolides, e.g., for amphotericin B in *Streptomyces nodosus* (AmphRIV; Carmody et al., 2004), for filipin in *Streptomyces avermitilis* (PteF; Omura et al., 2001; Ikeda et al., 2003) and *Streptomyces filipinensis* (FilF; Payero et al., 2015), and for candicidin in *Streptomyces griseus* (FscRI; Chen et al., 2003). These positive regulators are required for the production of the respective antibiotics in the producer strains (Chen et al., 2003; Vicente et al., 2014). The sequences of these PAS-LuxR regulators are well conserved and there is cross-complementation of pimaricin production when the genes *amphRIV*, *pteF* or *nysRIV* are transferred to a *pimM* negative mutant of *S. natalensis* (Santos-Aberturas et al., 2011a). The effect of PimM in *S. natalensis*, and PteF in *S. avermitilis*, results from binding to a 16 nucleotides region with dyad symmetry and a consensus sequence of CTVGGGAWWTCCCBAG (Santos-Aberturas et al., 2011a; Vicente et al., 2015). Consequently, *Streptomyces albus*, *S. nodosus*, and *S. avermitilis* transformed with *pimM* resulted in increased production of candicidin, amphotericin and filipin, respectively (Santos-Aberturas et al., 2011a; Olano et al., 2014). Surprisingly, production of the polyketide/non-ribosomal peptide antimycin was also activated by PimM in *S. albus* (Olano et al., 2014).

*Streptomyces clavuligerus* is the industrial producer of clavulanic acid (CA) (Baggaley et al., 1997; Liras et al., 2008), a β-lactamase inhibitor that is used clinically in combination with β-lactam antibiotics. The *S. clavuligerus* genome contains 49 annotated biosynthetic gene clusters (Medema et al., 2010) for the production of secondary metabolites, including CA, cephamycin C, holomycin and naringenin (Liras, 1999, 2014; Álvarez-Álvarez et al., 2015). However, most of these secondary metabolite gene clusters are cryptic, i.e., no information about the chemical structures of their products are available, or are silent and therefore not expressed in the media and conditions used thus far to grow *S. clavuligerus*.

The use of heterologous transcriptional activators is a promising route to activate silent gene clusters to trigger the production of numerous secondary metabolites (Martín and Liras, 2015). Given the functional conservation of PAS-LuxR regulators across different strains, and their capacity to cross-regulate the production of polyene antibiotics and other structurally different compounds (Olano et al., 2014; Vicente et al., 2015), we introduced the *pimM* gene of *S. natalensis* into *S. clavuligerus* and studied its effect on both the transcription of biosynthetic pathways and the production of secondary metabolites.

## Materials and Methods

### Strains and Culture Conditions

The wild-type strain *S. clavuligerus* ATCC 27064 and its derived strain *S. clavuligerus*::pIB139, which has plasmid pIB139 (Wilkinson et al., 2002) integrated into the chromosome, were used as control strains. *S. clavuligerus*::*pimM* derives from the wild type strain and contains an integrated pCPpimM plasmid. This plasmid is a pIB139 derivative that carries the *pimM* gene under the control of the constitutive *ermE*^∗^ promoter (Santos-Aberturas et al., 2012). Both pIB139 and pCPpimM were introduced by conjugation from *Escherichia coli* ET12567/pUZ8002 into *S. clavuligerus*, and integration into the genome was confirmed by analysis of apramycin resistance and by PCR amplification of the *aac3(IV)* and *pimM* genes (Supplementary Table S1), respectively.

*Streptomyces clavuligerus* strains were precultured in trypticase soy broth (TSB) for 24 h at 28°C with shaking at 220 rpm until the 1/10 diluted culture reached an optical density (OD_600_) of around 0.65. These seed cultures were used to inoculate (5%, v/v) duplicate or triplicate 500 ml triple-baffled flasks containing 100 ml of liquid medium, and cultures were grown for 96 h under the same conditions. The following liquid media were used: TBO (Higgens et al., 1974), ME (Sánchez and Braña, 1996), MEY (Kieser et al., 2000), MS (Hobbs et al., 1989), ISP4 (Difco^TM^) (Shirling and Gottlieb, 1966), TSA (Sambrook and Russell, 2001), SA (Aidoo et al., 1994), 2xTY (Sambrook and Russell, 2001), modified R5 (Thompson et al., 1982), and modified R2YEG (R2YEGm) (Thompson et al., 1980) both of them lacking sucrose and glucose and carrying maltose as carbon source. When required, the seed cultures were grown in TSA plates, which, after 24-h growth, were used to inoculate various solid media (TBO, ME, MEY, MS, ISP4, mR2YEG, 2xTY, mR5, TSA, and SA) to determine antifungal activity. SA medium (Aidoo et al., 1994) was used to determine clavulanic acid and cephamycin C production levels. ME medium (Sánchez and Braña, 1996) was used to analyze aerial mycelium formation and sporulation of the strains. Apramycin (50 μg/ml) and nalidixic acid (25 μg/ml) were added to cultures as required.

### Antibiotic Assays

Clavulanic acid and cephamycin C were quantified as indicated by Pérez-Redondo et al. (1998).

Tunicamycin complex standard from *Streptomyces* sp. (Abcam Biochemicals) was used as the source for the isolation of individual tunicamycins (see below).

#### Detection of the Antifungal Activity

*Streptomyces clavuligerus*::*pimM* and *S. clavuligerus*::pIB139 were cultured in the 10 solid media described above for 96 h. The metabolites produced were extracted from the agar (25 ml) with 60 ml of methanol, the culture-methanol mixtures were shaked for 15 min, filtered through filter paper and concentrated in a SpeedVac to 1 ml volume. The mycelium from MEY liquid culture (100 ml) was centrifuged and extracted with 30 ml of methanol and the MEY culture supernatant (100 ml) was mixed with an equal volume of methanol, treated as indicated above and concentrated to 2 ml final volume. Antifungal activity was detected by bioassays of 40 μl of the concentrated extracts with *Candida utilis* CECT 1061 (Antón et al., 2007). Amphotericin B was used as a positive control of antifungal activity.

### Metabolic Profiling and Characterization of the Antifungal Compound

#### Sample Preparation and LC-MS Analysis

Three independent cultures of each strain (*S. clavuligerus*::*pimM* and *S. clavuligerus*::pIB139) were grown in 100 ml MEY liquid medium for 96 h at 28°C with 220 rpm shaking. The mycelium of 50 ml of each culture was isolated by centrifugation, mixed with 15 ml of methanol and shaked for 15 min. The mycelium methanolic extracts were filtered through filter paper and concentrated in a SpeedVac to 1 ml. Bioassays against *C. utilis* were performed with 40 μl of the concentrated methanolic extracts to verify antifungal activity. A methanolic extract from fresh MEY media was used as a negative control. For liquid chromatography – mass spectrometry (LC-MS) analysis, the mycelium methanolic extracts were further concentrated in a SpeedVac to 100 μl and analyzed using a Shimadzu Nexera X2 UHPLC coupled to a Shimadzu IT-TOF mass spectrometer. Samples (5 μl) were injected onto a Phenomenex Kinetex 2.6 μ C18 column (50 mm × 2.1 mm, 100 Å) set at 40°C, eluting over 6 min with a linear gradient of 5–95% acetonitrile in water containing 0.1% formic acid at a flow-rate of 0.6 ml min^−1^. Positive and negative mode mass spectrometry data were collected between *m/z* 200 and 2000, and MS^2^ data were collected using collision-induced dissociation, with an exclusion time of 1 s for a given species, where MS-MS^2^ cycle period was 0.26 s. The obtained data were statistically compared using Profiling Solution 1.1 (Shimadzu), with an ion *m/z* tolerance of 100 mDa, a retention time tolerance of 0.1 min and an ion intensity tolerance of 100,000 units, and manually filtering the signals that corresponded to the culture medium components.

#### Semi-Preparative Fractionation

The mycelium extract of a 150 ml MEY culture of *S. clavuligerus*::*pimM* was vacuum dried, resuspended into 100 μl of 50% methanol/water and injected onto a Phenomenex Luna 5 μm C18(2) column (250 mm × 10 mm, 100 Å), eluting at a flow-rate of 4 ml min^−1^ with a gradient of (A) acetonitrile and (B) water with 0.1% formic acid, as follows: 0–1 min 5% B; 22 min 95% B, 22–24 min 95% B, 24.1 min 5% B, 24.1–27 min 5% B. Samples (4 ml) were collected every minute and were then dried using a SpeedVac and resuspended in 150 μl of 50% methanol for further LC-MS and antifungal activity analyses, as described above. The same HPLC method and downstream sample processing were employed for the fractionation of 1 mg of tunicamycin standard (diluted in 500 μl of 50% dimethyl sulfoxide, DMSO, in water) for the isolation and characterization of individual tunicamycins, resulting in the separation of three pure tunicamycins (*m/z* 815.39, 829.41 and 857.44). On the basis of their original LC-MS peak areas, the concentrations of these samples were adjusted with the addition of solvent to reach the same levels (8,000 peak area units/μl) in all cases. Two-fold serial dilutions of these three equally concentrated tunicamycins where then employed in bioassays against *C. utilis*.

#### Mass Spectral Molecular Networking

LC-MS spectra were obtained using the same LC-MS parameters as defined above, where MS^2^ data were collected in a data-dependent manner for the most abundant species between *m/z* 200 and 2000, with an exclusion time of 1 s for a given species, where the MS-MS^2^ cycle period was 0.26 s. LC-MS^2^ data from triplicate cultures of *S. clavuligerus*::pIB139 and *S. clavuligerus*::*pimM* were used to construct mass spectral networks using the Global Natural Products Social Molecular Networking server (GNPS^1^) (Nguyen et al., 2013; Wang M. et al., 2016). Here, the data were clustered with MS-Cluster with a parent mass tolerance of 0.5 Da and a MS^2^ fragment ion tolerance of 0.5 Da to create consensus spectra. The following settings were used for network construction: minimum pair cosine = 0.65, minimum matched fragment ions = 3, minimum cluster size = 2, minimum peak intensity = 25. The spectra in the resulting networks were then searched against all GNPS spectral libraries available at the time of analysis. Raw results are available at the GNPS server^2^. Matches were made between network spectra and library spectra where a score was above 0.7 and there were at least 3 matched peaks. Network data were visualized using Cytoscape 3.5.1. Nodes were removed when erroneously duplicated by GNPS (same retention time, mass, and MS^2^ spectrum). The peak areas of extracted ion chromatograms for each node in the tunicamycin network were calculated using Shimadzu Quant Browser and processed with Microsoft Excel before being graphically integrated into the Cytoscape-generated network as the average of the calculated areas for the triplicate cultures.

### Nucleic Acid Isolation, PCR and qPCR

DNA was isolated as described by Pospiech and Neumann (1995). RNA sampling was carried out at 84, 90, and 96 h. Nucleic acid isolation and integrity analysis were performed as indicated by Martínez-Burgo et al. (2015).

The PCR reactions were performed as described by Kieser et al. (2000) using a T-gradient thermocycler (Biometra) and the oligonucleotide primers shown in Supplementary Table S1. The PCR reaction contained in 20 μl volume: 40 ng DNA template, 0.2 mM each deoxynucleoside triphosphate (dNTP), 4% DMSO, and 1 unit of Taq DNA Polymerase (Kapa Biosystems). The amplification was carried out with an initial 3 min 95°C denaturing step. The cycles comprised a denaturing step of 30 s at 95°C, annealing for 30 s at the corresponding temperature (Supplementary Table S1) and extension for 2 min at 72°C. The amplification was completed with a final extension of 5 min at 72°C. Quantification and purity analysis of PCR products was determined using a NanoDrop ND 1000 UV–vis (Thermo Scientific) and the fidelity of the amplified products was confirmed by sequencing.

Plasmids pSCL1, pSCL2, and pSCL4 were detected and quantified by qPCR using 20 ng DNA as described by Lee et al. (2006). The following genes were analyzed: to detect pSCL1, SclaA2_010100027570, and SclaA2_010100027590; for pSCL2, SclaA2_010100027690 and SclaA2_010100027930; and for pSCL4, *brp* and *parB*_*pSCL*4_. The chromosomal genes *adpA* and *hrdB* (named *rpoD* in *S. clavuligerus*) were used as controls. The oligonucleotides used were described by Álvarez-Álvarez et al. (2014) and are included in Supplementary Table S1.

### RT-qPCR

Gene expression analysis and quantification was performed by RT-qPCR as described by López-García et al. (2010) using the 2^−ΔΔCt^ method (Schmittgen and Zakrajsek, 2000; Livak and Schmittgen, 2001), and the constitutive housekeeping gene *hrdB* gene as internal control (Aigle et al., 2000). cDNAs for RT-qPCR analysis were synthesized as described by López-García et al. (2010). Negative controls were always carried out to confirm the absence of DNA contamination. RT-qPCR oligonucleotide primers are shown in Supplementary Table S1.

### Microarray Design

The microarrays used in this work have been previously described by Martínez-Burgo et al. (2015). RNA was extracted from the culture samples at 84, 90, and 96 h and the analysis was performed for two biological replicates for each condition (two strains and three growth times). Labeling of RNA preparations with Cy3-dCTP, labeling of genomic DNA as the reference sample with Cy5-dCTP (4 pmol/50 μl hybridization solution), and the purification procedures were carried out as described previously (Álvarez-Álvarez et al., 2014). The hybridization conditions, washing, scanning with Agilent Scanner G2565BA, and the quantification of the images were carried out as previously described (Sartor et al., 2004; Rodríguez-García et al., 2007; Yagüe et al., 2014).

### Transcriptome Analysis

Transcriptome analysis using microarrays was performed as indicated by Martínez-Burgo et al. (2015). The *M_g_* transcription values obtained are proportional to the abundance of the transcript for a particular gene (Mehra et al., 2006) and correspond to the transcription values of the six experimental conditions, mutant versus control, corresponding to the three studied growth times. For each gene, *M_c_* values and *P*-values were calculated (three sets of values, one for each comparison). The *M_c_* values are the binary log of the differential transcription between the mutant and the control strain. The Benjamini–Hochberg (BH) false-discovery rate correction was applied to the *P*-values. For each comparison, a result was considered statistically significant if the BH-corrected *P*-value was ≤0.1. A positive *M_c_* value indicates upregulation, and a negative *M_c_* value indicates downregulation. 75 genes were found to be statistically significant with *M_c_* value ≤ −1 or M_*c*_ value ≥ 1 in some of the three sampling times (Supplementary Table S2).

### Microarray Data Accession Number

The microarray data used in this work have been deposited in the National Center for Biotechnology Information-Gene Expression Omnibus database under accession number GSE87092.

### Bioinformatic Analysis of PimM-Binding Sites and DNA-Protein Binding Assays

A bioinformatic search of potential sequences within the *S. clavuligerus* genome that could be recognized by PimM was performed using the PimM_AR02DyR frequency matrix (Ordóñez-Robles et al., 2016) and the matrix-scan tool of the RSAT server (Medina-Rivera et al., 2015). The PimM frequency matrix summarizes the nucleotide composition of the PimM binding sites that have been previously defined by DNase I footprinting (Santos-Aberturas et al., 2011b). The information content of each sequence (*Ri* value) was calculated according to Schneider (1997).

Interaction between PimM and its putative target sequence was studied by electrophoretic mobility shift assays (EMSAs) performed as described by Santos-Aberturas et al. (2011a) using pure GST-PimM. The DNA fragment used for EMSAs was amplified by PCR using the primers ccaR_cmcH F and ccaR_cmcH R (Supplementary Table S1) and *S. clavuligerus* chromosomal DNA as template. A probe containing the *pimS1*-*pimD* intergenic region was also obtained by PCR using the primers pimS1_pimD F and pimS1_pimD R (Supplementary Table S1) and *S. natalensis* chromosomal DNA as template. Amplification product was sequenced to discard the presence of any mutations and then labeled at both ends with digoxigenin with DIG Oligonucleotide 3′-End Labeling Kit, 2nd Generation (Roche Applied Science). A standard binding reaction contained 0.025 ng/μl of labeled DNA probe, 40 mM Tris-HCl, pH 8.0, 0.4 mM MgCl_2_, 5 mM KCl, 0.1 mM DTT, 7.8 mM glutathione, 0.005% Nonidet P-40, 50 μg/ml poly(dI-dC), 20.6% glycerol and variable concentrations of protein. The reactions were performed at 30°C for 10 min and then loaded onto a 5% polyacrylamide (29:1) native gel in 0.5X TBE buffer. After electrophoresis (1.5 h, 70 V, 4°C), DNA was transferred onto a nylon membrane (HyBond-N, Amersham Biosciences) in 0.5X TBE buffer (30 min, 200 mA). The DNA was fixed by UV crosslinking, detected with anti-digoxigenin antibodies, and developed by chemiluminescence with CDP-Star^TM^ reagent (Roche Applied Science).

## Results

### Characterization of *S. clavuligerus*::*pimM*

A single copy of *pimM* was introduced in the *S. clavuligerus* genome using the pIB139-based integrative plasmid pCPpimM, where expression of the *pimM* gene is under the control of the constitutive *ermE^∗^* promoter. Expression of *pimM* in *S. clavuligerus*::*pimM* was analyzed by RT-qPCR using *S. clavuligerus*::pIB139 as a negative control and the *rpoD* chromosomal housekeeping gene of *S. clavuligerus* as a reference gene. This showed a *pimM* signal in *S. clavuligerus*::*pimM* of about 80-fold above the background signal in *S. clavuligerus*::pIB139, indicating that *pimM* is expressed in *S. clavuligerus*::*pimM* (Supplementary Figure S1A). The relative amount of DNA of linear plasmids pSCL1, pSCL2, and pSCL4, in *S. clavuligerus*::pIB139 and *S. clavuligerus*::*pimM*, were compared by qPCR with those from the wild type strain *S. clavuligerus* ATCC 27064. This confirmed that the three strains contain a similar number of the three plasmids (Supplementary Figure S1B).

### Transcriptomic Analysis of *S. clavuligerus*::*pimM* in Comparison to the Control Strain *S. clavuligerus*::pIB139

A microarray-based transcriptomic analysis of *S. clavuligerus*::*pimM* and the control strain was performed on 84, 90, and 96 h MEY-grown culture samples. The transcriptomic data were filtered at one of the three sampling times applying the criteria: *M_c_* ≥ 1.0 or *M_c_* ≤-1.0, and a BH-corrected *P*-value ≤ 0.1, to restrict the data to only those genes whose expression level were highly and significantly affected by PimM at least at one of the three sampling times. While many other genes were up- or downregulated (see below), only 18 genes were upregulated and 58 genes were downregulated in *S. clavuligerus*::*pimM* according to these criteria (Supplementary Table S2).

#### Secondary Metabolite Gene Clusters Affected by PimM

Analysis of the transcriptome of the 49 annotated secondary metabolite gene clusters (SMCs) described in *S. clavuligerus* (Chen et al., 2010; Medema et al., 2010) showed that expression of *pimM* resulted in the upregulation of three gene clusters (SMC10, SMC11, and SMCp5), downregulation of two gene clusters (SMC22 and SMCp25), and the up- or downregulation of blocks of genes in four clusters (SMC6, SMC13, SMC14, and SMCp6).

**FIGURE 1:**
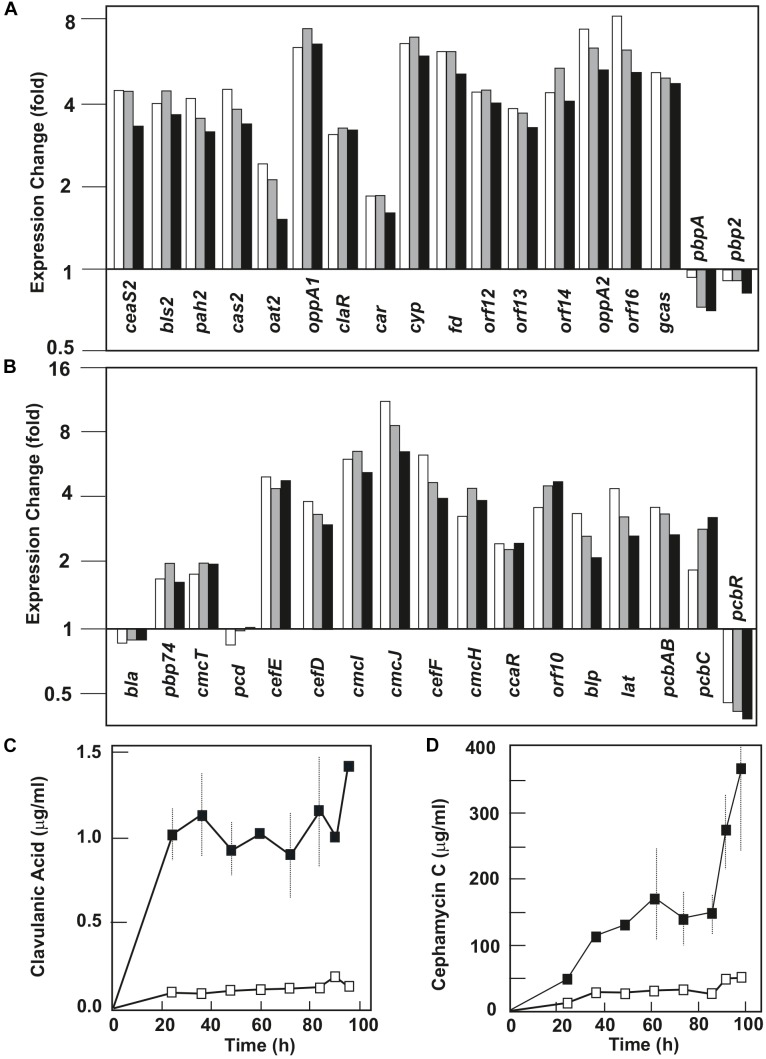
Effect of PimM on the transcription level of the clavulanic acid and cephamycin C gene clusters and on the production of these two metabolites. Change in the expression level of genes for clavulanic acid **(A)** and cephamycin C **(B)** biosynthesis in *Streptomyces clavuligerus*::*pimM*. The columns represent the average of the expression fold-change at 84 h (white columns), 90 h (gray columns), and 96 h (black columns). Gene names are indicated at the bottom of the columns. Expression level values of *S. clavuligerus*::*pimM* are compared to those of the control strain, *S. clavuligerus*::pIB139, normalized as 1. Clavulanic acid **(C)** and cephamycin C **(D)** production in MEY medium in *S. clavuligerus*::*pimM* (black squares) and *S. clavuligerus*::pIB139 (white squares). Vertical lines indicate standard deviations from three biological replicates.

**FIGURE 2:**
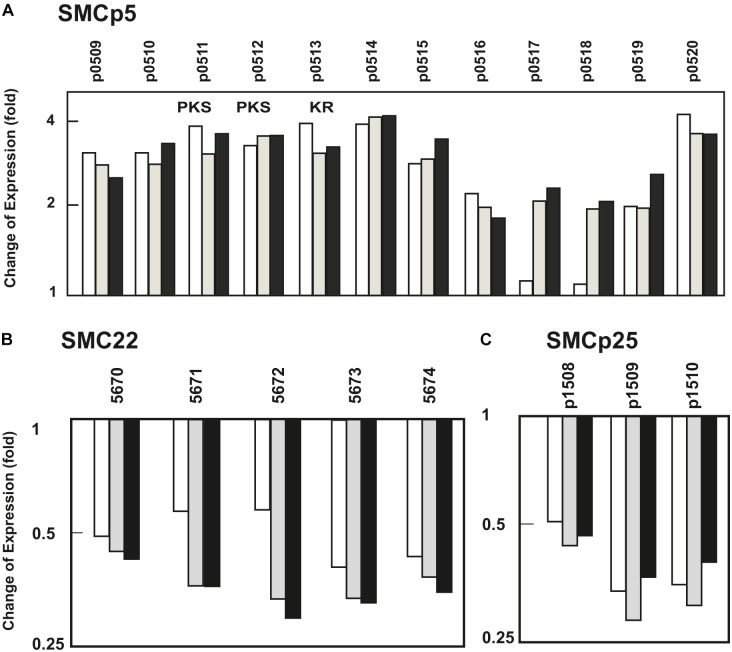
Effect of PimM on the transcription levels of SMCp5, SMC22, and SMCp25. Change in the expression level of genes of **(A)** SMCp5, an upregulated gene cluster that encodes a PKS pathway, and two downregulated gene clusters, **(B)** SMC22 and **(C)** SMCp25, predicted to encode a terpene-like compound, and a clavam-like β-lactam, respectively. In the panels the columns represent the average of the expression fold-change at 84 h (white columns), 90 h (gray columns), and 96 h (black columns). Numbering of the SCLAV genes is indicated. Expression level values of *S. clavuligerus*::*pimM* are compared to those of the control strain, *S. clavuligerus*::pIB139, normalized as 1. Genes encoding polyketide synthases and ketoreductases are indicated by the letters PKS and KR, respectively.

SMC10 and SMC11 are responsible for the biosynthesis of the clinically important β-lactams clavulanic acid and cephamycin C, respectively. These clusters were upregulated in all their biosynthetic and major regulatory genes (*ccaR*, *claR*) (Figure 1 and Supplementary Table S5), and form a contiguous supercluster that is discussed below in more detail. Cluster SMCp5 (SCLAV_p0509 to p0520) encodes a type II polyketide synthase pathway. All genes in this cluster showed an increase in expression level between two and fourfold at 90 and 96 h in *S. clavuligerus*::*pimM* versus the control strain (Figure 2A and Supplementary Table S5).

In cluster SMC22 (SCLAV_5670-5674), predicted to be involved in the formation of a terpene-like compound, all genes were downregulated between 1.7- and 3-fold in all three sampling times (Figure 2B and Supplementary Table S5). The three genes in cluster SMCp25 (SCLAV_p1508 to p1510), which might produce a clavam-type β-lactam, were downregulated between 2- and 3.3-fold in the three sampling times (Figure 2C and Supplementary Table S5).

Some gene cluster showed blocks of upregulated and/or downregulated genes. In the biosynthetic gene cluster SMC6 for the siderophore desferrioxamine, a block of genes (SCLAV_1948 to SCLAV_1951) was upregulated between 2.2- and 3.4-fold (Supplementary Figure S2A). These four genes are orthologous to *desABCD*, genes essential for the biosynthesis of desferrioxamine in *Streptomyces coelicolor* (Barona-Gómez et al., 2004). In contrast, the adjacent genes (SCLAV_1942 to SCLAV_1946) were downregulated between 1.7- and 3.4-fold.

The SMC13 gene cluster is predicted to produce a hybrid polyketide-non-ribosomal peptide and genes SCLAV_4461-4462 and SCLAV_4464-4472 were upregulated between 2- and 7.2-fold in the three sampling times (Supplementary Figure S2B). SCLAV_4466, which encodes a trimodular hybrid polyketide synthase - non-ribosomal peptide synthetase (PKS-NPRS), was upregulated fourfold with respect to the control strain.

In SMC14, which encodes a large multimodular NRPS, 6 of its 19 genes (SCLAV_4751–4753 and SCLAV_4755-4757), were upregulated between two and fourfold. However, the expression levels of all NRPS genes (SCLAV_4742, SCLAV_4749, SCLAV_4750, and SCLAV_4758) were unaffected (Supplementary Figure S2C).

The SMCp6 gene cluster (SCLAV_p0563 to p0588) may produce a hybrid NRPS-PKS-terpene (or separate compounds). PimM expression led to the downregulation of 6 from its 24 genes (SCLAV_p0563 to p0568), with a change of expression level between 2.5- and 25-fold (Supplementary Figure S2D). This block of genes includes SCLAV_p0564, which encodes a NRPS, SCLAV_p0566 (a PKS-like ketosynthase), and SCLAV_p0568 (an IclR-family transcriptional regulator).

##### Overexpression of the clavulanic acid and clavam gene clusters

PimM had a positive effect on the expression level of the clavulanic acid gene cluster (SMC10, Figure 1A and Supplementary Table S5), where all clavulanic acid biosynthetic genes were upregulated between 1.5- and 8-fold in the three sampling times. This corresponded with an observed overproduction of this clinically important β-lactamase inhibitor in *S. clavuligerus*::*pimM*, where production was 1.4 μg/ml, 10-fold higher than in *S. clavuligerus*::pIB139 (Figure 1C). Genes encoding the early steps of the pathway (*ceaS2*, *bls2*, *pah2*, and *cas2*) were overexpressed between 3- and 4-fold; genes for late steps of the pathway (*gcas*, *orf16*, *orf14*, *orf13*, *orf12*, *cyp*-*fd*, and *car*) were overexpressed between 1.6- and 8-fold. The genes *oppA1* and *oppA2*, encoding oligopeptide permeases essential for CA biosynthesis (Lorenzana et al., 2004), showed a clear increase in expression levels (seven and sixfold, respectively). In contrast, genes of the related clavam gene cluster (SMC9, SCLAV_2921 to 2935), encoded distantly from SMC10, and of the CA paralogous, pSCL4-situated, gene cluster (SMCp13, SCLAV_p1069 to p1082), showed no significant differences in their expression levels (not shown).

##### Overexpression of the cephamycin C gene cluster

PimM overexpression led to the upregulation of almost every gene in the gene cluster of the β-lactam antibiotic cephamycin C (SMC11, Figure 1B and Supplementary Table S5). All genes in this cluster were upregulated between 1.6- and 11-fold in the three sampling times, except *pcd* and *bla*, which were unaffected, and *pcbR* (a β-lactam resistance gene), which was downregulated 2.5-fold. Gene cluster overexpression corresponded with the overproduction of cephamycin C by *S. clavuligerus*::*pimM*, which was 7 times higher than in *S. clavuligerus*::pIB139 (Figure 1D) and led to the production of 360 μg/ml of cephamycin C after 96 h fermentation. The most highly upregulated genes were *cmcI* and *cmcJ* (6- and 11-fold, respectively), encoding a two-protein complex that catalyzes late-stage methoxylation in the cephamycin C pathway, and can be used to convert cephalosporins into 7α-methoxycephalosporins (Enguita et al., 1996; Shao et al., 2014). The cephamycin C-clavulanic acid genes *pbpA*, *pbp2*, and *pcbR*, located in the supercluster and predicted to be involved in β-lactam resistance, were downregulated. However, the resistance genes *pbp74*, *cmcT*, *orf10*, and *blp* were slightly overexpressed (Figure 1B and Supplementary Table S5).

##### GST–PimM binds to the intergenic region *ccaR*-*cmcH*

**FIGURE 3:**
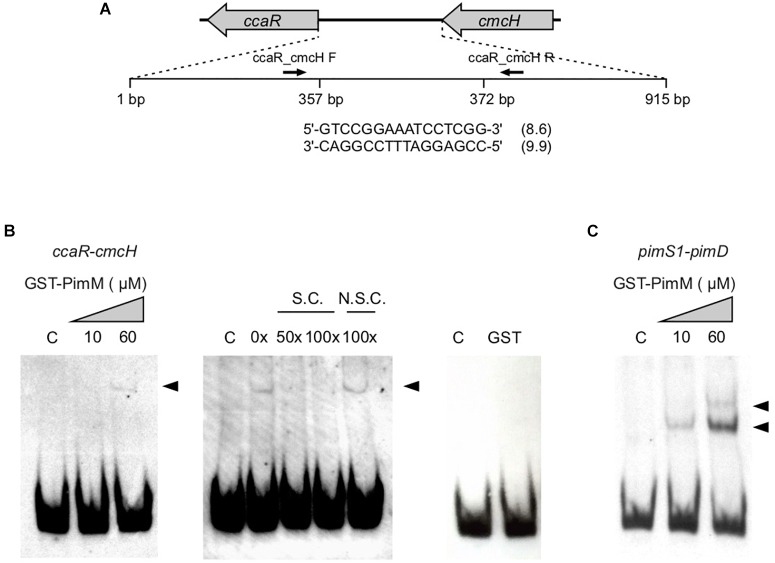
GST-PimM DNA binding assay. **(A)** Schematic representation of the intergenic region *ccaR*-*cmcH* located in the cephamycin C cluster. The nucleotide sequence of the putative PimM-binding site is shown below and the information content (*Ri*) in bits is indicated in parentheses. Sizes are not proportional. **(B)** Binding of pure GST-PimM to the intergenic region *ccaR*-*cmcH* as shown by EMSAs. The DNA fragment was mixed with increasing concentrations of GST-PimM (0–60 μM) (left panel). The central panel shows a competition experiment between labeled and growing concentrations of unlabelled probe. Right panel, control reaction with 60 μM pure GST protein. C, reaction without protein; N.S.C., non-specific competitor DNA; S.C., specific competitor DNA. DNA-protein complexes are indicated by arrowheads. **(C)** Binding of pure GST-PimM to the intergenic region *pimS1-pimD* from *S. natalensis* (containing two PimM-target sequences) as shown by EMSAs. The reaction conditions were the same as for the *ccaR-cmcH* probe.

Given the increase of both clavulanic acid and cephamycin C production in *S. clavuligerus*::*pimM* in relation with the control strain *S. clavuligerus*::pIB139, an *in silico* search of PimM binding sites within the *S. clavuligerus* genome was performed as described in Section “Materials and Methods.” A putative binding site for PimM (*Ri* values of 8.6 and 9.9 for the direct and reverse strands, respectively) was identified in the intergenic region between *ccaR* and *cmcH* of the cephamycin C gene cluster (Figure 3A). In order to confirm the binding of PimM to this region, EMSAs were performed with pure GST-PimM and a labeled 80 bp fragment containing its putative binding sequence. Results showed the appearance of a retarded band (Figure 3B), in agreement with the presence of a PimM box in this region. This band disappeared by the addition of the same unlabelled DNA, but not by non-specific competitor DNA, thus confirming that the interaction was specific (Figure 3B). A control reaction with pure GST showed no interaction, discarding the binding of this protein to the region (Figure 3B). An additional control was performed with a 308 bp probe containing the *pimS1*-*pimD* intergenic region from *S. natalensis*. Two retardation bands, corresponding to the two PimM target sites present in this region, were detected (Figure 3C). It is interesting to note that there is still unbound DNA in the reactions with 60 μM protein, indicating that the interaction of GST-PimM with its targets is rather weak. The affinity of PimM for its native sites is stronger than for the one located in the *ccaR* promoter, which is probably due to their higher Ri values (Santos-Aberturas et al., 2011b).

#### Genes With Diverse Functions

In addition to genes in SMC10, SMC11, SMC13, and SMCp6, the expression of 63 genes was statistically affected in *S. clavuligerus*::*pimM* compared to the control strain (Supplementary Table S2). This included five additional regulatory proteins, including downregulation of a RNA polymerase sigma U factor (SCLAV_2070). The remaining genes are predicted to be involved in a variety of cellular functions. In particular, two blocks of divergent genes, SCLAV_0832-0834 and SCLAV_0835-0837, were significantly downregulated, between 3- and 14-fold. These genes encode a short chain dehydrogenase, a phenylacetaldehyde dehydrogenase, a glutamine synthetase, a regulatory protein, an ethanolamine transporter and an amidotransferase, respectively, and may be involved in nitrogen metabolism. Some of these genes might not be affected directly by PimM but by cascade mechanisms involving other regulators, such as CcaR.

#### Validation of the Transcriptomic Data

Microarray transcriptomic data were validated using RT-qPCR with the same RNA samples used for the transcriptomic studies at 90 h. Twelve genes were validated, including genes involved in the biosynthesis of clavulanic acid, cephamycin C, desferrioxamine, SMC13, SMCp5, SMCp6, two genes encoding regulatory proteins, and two genes encoding proteins with diverse functions (Supplementary Figure S3A). The high correlation between RT-qPCR and microarray data (Pearson correlation coefficient = 0.98) strongly supports the results obtained in the microarrays (Supplementary Figure S3B).

#### Other Secondary Metabolites Produced by *S. clavuligerus*::*pimM*

**FIGURE 4:**
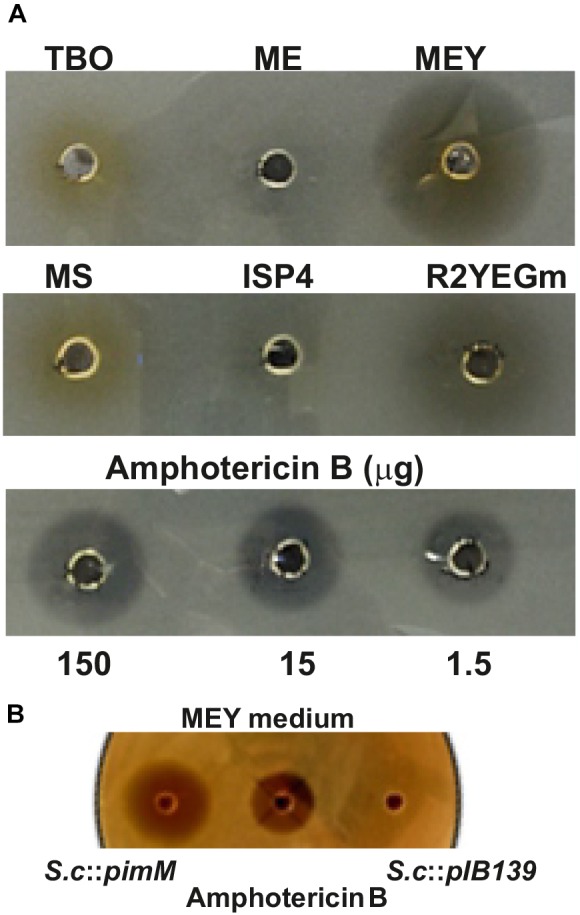
Bioactivity of *S. clavuligerus*::*pimM* and *S. clavuligerus*::pIB139 extracts against *C. utilis*. **(A)** Bioassay of concentrated extracts from *S. clavuligerus*::*pimM* grown on TBO, ME, MEY, MS, ISP4, and mR2YEG solid media. The media assayed are indicated on top of the bioassay wells. Amphotericin B (150, 15, and 1.5 μg) was used as bioassay positive control. **(B)** Bioassay of concentrated extracts from *S. clavuligerus*::*pimM* (left) and *S. clavuligerus*::pIB139 (right) grown in MEY medium and amphotericin B (150 μg) used as bioassay positive control (center).

The transcriptomic data indicated that PimM expression affected the regulation of a number of biosynthetic gene clusters in addition to clavulanic acid and cephamycin C. Therefore, to assess the production of bioactive compounds in *S. clavuligerus*::*pimM* that were silent in *S. clavuligerus*::pIB139, these two strains were separately cultured in 10 solid media for 96 h. Extracts from the mycelium and medium of each plate were assayed for inhibitory activity against *E. coli* Ess22-31, *Micrococcus luteus* and *C. utilis*. There were no differences between the diameters of the zones of inhibition of extracts from *S. clavuligerus*::*pimM* and the control strain in the bioassays against bacteria (not shown). However, when cultured in MEY and mR2YEG, a zone of inhibition against *C. utilis* was detectable in extracts of *S. clavuligerus*::*pimM* (Figure 4A). This activity was absent in MEY cultures and was very weak in mR2YEG cultures of *S. clavuligerus*::pIB139 (Figure 4B). No antifungal activity was detected in cultures grown in other media. The antifungal activity from the mycelium and the culture supernatant of MEY liquid cultures was measured separately and detected only in the mycelial extract.

##### Metabolic profiling of the *pimM* expression effects in *S. clavuligerus* and identification of the antifungal compound

The notable increase in the production of clavulanic acid and cephamycin C, together with the variety of transcriptional changes induced by the expression of *pimM* and the triggering of antifungal activity, prompted an analysis of the global metabolic effects of PimM expression. Untargeted metabolic profiling of LC-MS data was carried out to compare *S. clavuligerus*::*pimM* and *S. clavuligerus*::pIB139. A comparative analysis of mycelial extracts (Supplementary Tables S3, S4) showed significant differences in the metabolic profiles of these strains. To check whether any differential peaks were responsible for the antifungal activity of *S. clavuligerus*::*pimM*, the extracts were fractionated using semi-preparative HPLC and assayed against *C. utilis*. The antifungal activity was localized in two fractions, and further LC-MS analysis (Supplementary Figure S4) revealed the presence of some metabolites that had been identified as being overproduced in *S. clavuligerus*::*pimM* in comparison to *S. clavuligerus*::pIB139 (Supplementary Tables S3, S4). The *m/z* values of these metabolites were 582.30, 596.32, 610.33, and 624.34 in positive ion detection mode, and 801.38, 815.39, 829.40, and 843.42 in negative mode.

**FIGURE 5:**
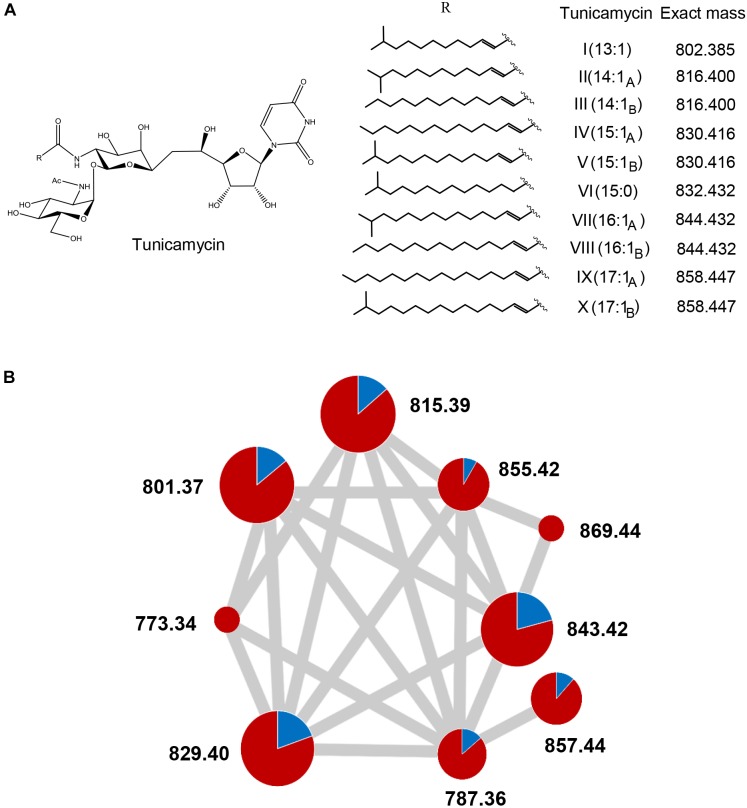
Structure of tunicamycins and overproduction in *S. clavuligerus::pimM*. **(A)** Structure and diversity of known tunicamycins. **(B)** Tunicamycin molecular networking analysis showing a network corresponding to the tunicamycins detected in *S. clavuligerus::*pIB139 (blue) and *S. clavuligerus::pimM* (red). The circles situated in the nodes of the network represent the relative amounts of each tunicamycin congener in each strain. The three different circle sizes arbitrarily represent dominant, intermediate and minor levels of abundance within the tunicamycin complex (accurate absolute ion count values are shown in Supplementary Figure S6). The gray edges joining the nodes correspond to the existence of shared MS^2^ signals, as identified by GNPS (Wang M. et al., 2016).

These compounds were unequivocally identified as different members of the tunicamycin complex (Takatsuki et al., 1971) based on their masses (Figure 5) and MS^2^ fragmentation (Supplementary Figure S5) (Tsvetanova and Price, 2001). Additionally, the comparison to a commercial tunicamycin standard was performed for confirmation of the observed MS^2^ fragmentation pattern (Supplementary Figure S5). MS molecular networking analysis (Wang M. et al., 2016) (Figure 5B, raw results^3^) showed that these tunicamycins were overproduced alongside a wider family of tunicamycins that are predicted to differ in the length of an *N*-acyl side chain (Figure 5A). Quantification of the MS peak areas corresponding to each tunicamycin revealed that the increases in their productions levels in *S. clavuligerus:*:*pimM* respect to the control strain were 6.2-fold (*m/z* 787.36), 6.1-fold (*m/z* 801.37), 6.3-fold (*m/z* 815.39), 4.1-fold (*m/z* 829.40), 3.8-fold (*m/z* 843.42), 10.6-fold (*m/z* 855.43), and 7.6-fold (*m/z* 857.44), as shown in Figure 5B and Supplementary Figure S6. In total, the global overproduction of the tunicamycin complex with respect to the control strain was 5.3-fold, including two previously undescribed putative tunicamycins (*m/z* 773.34 and 869.44 in negative ion detection mode), which correspond with *N*-acyl side chains of 11 and 18 carbons of length, respectively (Figure 5A). Another putative tunicamycin (*m/z* 787.36, predicted acyl chain of 12 carbons) had not been previously described (Kenig and Reading, 1979; Tsvetanova and Price, 2001; Chen et al., 2010), but was also detectable at low levels in the control strain (Figure 5B).

**FIGURE 6:**
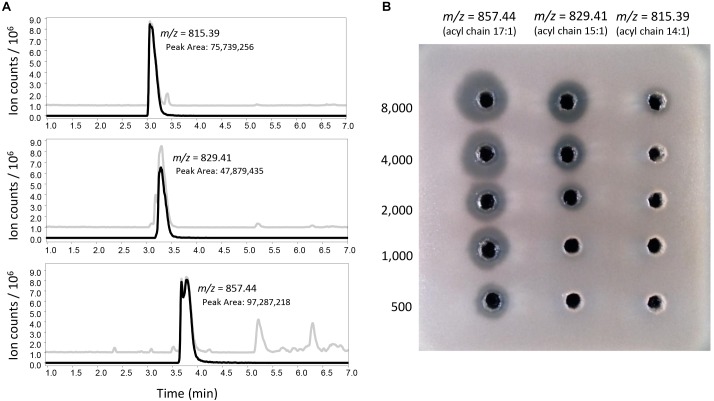
Antifungal activity of different isolated tunicamycins. **(A)** LC-MS analysis (negative mode) of tunicamycins purified from a commercial tunicamycin complex. Gray lines represent the base peak chromatogram for each sample. Black lines represent the extracted ion chromatogram corresponding to the mass of each tunicamycin congener. **(B)** Bioassays showing the relative antifungal activity of the purified tunicamycins. Each column corresponds to serial twofold dilutions of each purified tunicamycin. Concentrations are indicated in peak area units/μl, starting from 8,000 for the starting sample in each case.

The overproduction of the tunicamycins and their presence in the antifungal fractions suggested that they are responsible for the observed fungal inhibition, especially because the antifungal activity of the tunicamycin complex has been previously reported (Takatsuki et al., 1971). Interestingly, only the fractions containing significant amounts of tunicamycins with 15:1 and 16:1 acyl chains (*m/z* 829.4 and 843.42 in negative mode, Figure 5A and Supplementary Figure S4) exhibited antifungal activity, while fractions containing similar amounts of tunicamycins with shorter acyl chains (Figure 5A and Supplementary Figure S4) did were not antifungal, suggesting that tunicamycins carrying longer acyl chains are more active as antifungals. As this result was quite puzzling, and to discard the existence of a co-eluting antifungal, three pure tunicamycins (*m/z* 815.39, 829.41 and 857.44, Figure 6A) were tested for bioactivity against *C. utilis*. Serial dilutions revealed that the two tunicamycins with longer acyl chains indeed possess much higher antifungal activities (Figure 6B) and are 4–16 times more active than the tunicamycin carrying the shorter acyl chain. In contrast to the increase in expression of the clavulanic acid and cephamycin C gene clusters, expression of the tunicamycin gene cluster (SCLAV_4275-4287; not originally annotated as a SMC) was unchanged between *S. clavuligerus*::*pimM* and the control strain.

## Discussion

The PAS-LuxR type regulator PimM of *S. natalensis* was initially described as a pathway-specific transcriptional regulator of pimaricin biosynthesis (Antón et al., 2007; Santos-Aberturas et al., 2011a). Regulators of the PAS-LuxR class (AmphIV, NysRIV, and PteF) found in other polyene producing *Streptomyces*, were able to restore pimaricin production in *S. natalensis* Δ*pimM.* The *amphRIV* and *nysRIV* genes, encoding the PAS-LuxR regulators of *S. nodosus* and *S. avermitilis*, respectively, complemented pimaricin production when transferred to *S. natalensis pimM* mutants. In addition, introduction of a single copy of *pimM* into *S. nodosus* or *S. avermitilis* boosted the respective production of amphotericin and filipin. This suggests that these regulators are exchangeable, and their expression is a bottleneck in the biosynthesis of these polyenes (Santos-Aberturas et al., 2011a).

In addition to polyenes, the range of natural products pathways that are positively affected by these regulators has increased (Cheng et al., 2015). For example, production of the non-polyene polyketide oligomycin is activated in *S. avermitilis* by PteF (Vicente et al., 2015), and the production of the polyketide-non-ribosomal peptide antimycin in *S. albus* is increased when the heterologous *S. natalensis* PimM, or genes orthologous to PimM, were expressed in this strain (Olano et al., 2014; McLean et al., 2016).

In this study, we demonstrate that the expression of *S. natalensis* PimM in *S. clavuligerus* significantly increases the production of two clinically relevant β-lactam compounds, clavulanic acid and cephamycin C. The overexpression of the regulatory genes *claR* and *ccaR*, from 2- to 11-fold, and almost all biosynthetic genes of cephamycin C-clavulanic acid supercluster in *S. clavuligerus*::*pimM*, resulted in a 7- and 10-fold increase in production of cephamycin C and clavulanic acid, respectively, in comparison to the control strain (Figure 1C,D). These levels are similar to those observed in the industrial clavulanic acid production strain *S. clavuligerus* DS48802 obtained by random mutagenesis (two and eightfold compared to the wild-type strain) (Medema et al., 2011). Although with moderate affinity, the PimM regulator binds specifically to a DNA sequence upstream of *ccaR* (Figure 3). This mild affinity might be consequence of the weak interactions that GST-PimM establishes with its target DNA regions (Santos-Aberturas et al., 2011a). The binding of PimM to this promoter region explains the overexpression of *ccaR* that in turn leads to the upregulation of both cephamycin and clavulanic acid gene clusters and the overproduction of these two metabolites.

Multiple genes from across the *S. clavuligerus* genome were significantly affected (up- or down-regulated) by PimM expression, including a number of biosynthetic gene clusters, such as cluster SMCp5, in which all the genes were activated, or cluster SMC13, where nine genes were upregulated. It is possible that the products of these clusters were detected by untargeted metabolomics, where the production of numerous uncharacterized compounds was affected by PimM expression (Supplementary Tables S3, S4). However, the proportion of overproduced metabolites clearly outnumbers the observed transcriptomic changes observed, making any kind of linkage between the transcriptomic and metabolomic data highly speculative. This outnumbering could arise due to multiple congeners being formed from a single pathway, the formation of multiple adducts that can be detected by MS, or via indirect effects on the physiology of *S. clavuligerus*. Notably, some compounds were highly overproduced in *S. clavuligerus*::*pimM* and did not belong to a molecular network, such as *m/z* 669.33, which was detected in negative ion mode. Compounds like this are especially interesting for future characterization as potential products of the SMCs activated by PimM in *S. clavuligerus*.

Expression of PimM also activated the production of a compound with antifungal activity, which we showed is due to an increase in the production of the tunicamycin complex (Price and Tsvetanova, 2007). Tunicamycins are a group of nucleoside antibiotics that act as reversible inhibitors of translocase I in bacterial peptidoglycan biosynthesis and inhibit protein glycosylation in eukaryotic cells. Very recently, it has been shown that tunicamycins can be interesting scaffolds for the development of new antibiotics against tuberculosis (Dong et al., 2018). *S. clavuligerus* has been described as a producer of tunicamycins (Kenig and Reading, 1979) and PimM expression leads to a fivefold increase in tunicamycin production. Molecular networking revealed the existence of new tunicamycins whose masses strongly suggest a broader diversity of fatty acids incorporated through *N*-acylation than previously observed. It is very likely that Kenig and Reading (1979) did not reported these compounds because of their extremely low production levels in the wild type compared to *S. clavuligerus*::*pimM*. In addition to this, we observed that individual tunicamycins with longer acyl chains exhibit substantially higher antifungal activity, a finding in line with a previous report (Kamogashira et al., 1988). The stronger bioactivity of tunicamycins carrying longer acyl chains has been reported also in growth cone-mediated neurite extension (Patterson and Skene, 1994). Given this strong correlation between the bioactivity of tunicamycins and the length of their acyl chain, our discovery of new tunicamycin members with extreme acyl chain sizes (*m/z* 773.36 and 869.44 in negative ion detection mode) is especially interesting.

Surprisingly, tunicamycin overproduction was not accompanied by an increase in the expression of the tunicamycin gene cluster. There could be multiple possible explanations for this, such as an increase in the supply of tunicamycin precursors in *S. clavuligerus*::*pimM*, like 5- and 6-carbon carbohydrate precursors or uridine, that might constitute bottlenecks for tunicamycin biosynthesis. The absence of pathway-specific regulators in the tunicamycin SMC (Wyszynski et al., 2010) suggest that perhaps there are no specific signals triggering the biosynthesis of these compounds. Interestingly, there are 5 genes (out of a total of 14) in the tunicamycin SMC (*tunFGKLN*) that do not play a direct biosynthetic o resistance role but seem to play a part in ensuring adequate precursor supply (Widdick et al., 2018). These 5 genes have functional counterparts in primary metabolism and are not essential to produce tunicamycins, suggesting that other processes in the metabolic background of the cell can compensate their absence. In this scenario, it seems very plausible that the alteration of such metabolic background could directly impact the tunicamycins production levels. For example, our transcriptomic analyses have shown that there is a 5.5-fold increase in expression of SCLAV_4470, a gene encoding an extracellular lipase. This protein may hydrolyze lipids present in the medium thereby provide additional long chain fatty acids that could be incorporated as *N*-acyl substituents in the biosynthesis of tunicamycins. To explore this possibility, future work will determine the extracellular lipase activity and tunicamycins production levels of a strain featuring a SCLAV_4470 gene deletion.

The significant transcriptional changes resulting from expression of a heterologous pathway specific regulator may indicate similarity to a native transcriptional regulator. An *in silico* search in the *S. clavuligerus* genome for genes encoding PAS-LuxR regulators revealed one gene, SCLAV_0213, encoding a protein with 43% identity to PimM, which is especially high within their DNA-binding predicted domains (63% identity). SCLAV_ 0213 is located between clusters SMC4 and SMC5, which encode a terpene pathway and a type I PKS pathway, respectively (Medema et al., 2010), but it is not linked to either SMC. This PAS-LuxR regulator has not yet been characterized; it may specifically affect expression of SMC4 and SMC5 or may play a wider control of the secondary metabolism of *S. clavuligerus*. Whether PimM introduced into *S. clavuligerus* acts synergistically or independently from SCLAV_0213 is a matter for future studies.

In summary, our results show how PimM, the archetype of PAS-LuxR regulators, can modulate the biosynthesis of multiple SMCs when expressed in *S. clavuligerus*. Thus, PimM (and probably orthologous PAS-LuxR regulators) may be used in wild type and industrial *S. clavuligerus* strains to increase the production of cephamycin C, clavulanic acid and tunicamycins. The large increases reported here for these relevant bioactive compounds through a simple genetic modification is comparable to the previously reported for specific compounds by other laborious strategies (Zabala et al., 2013; Li et al., 2015; Lu et al., 2016; Wang Y. et al., 2016). Our work highlights the potential of heterologous expression of conserved transcriptional regulators as a biotechnological tool for strain improvement and drug discovery.

## Data Availability

The datasets generated for this study can be found in National Center for Biotechnology Information-Gene Expression Omnibus database under accession number GSE87092.

## Author Contributions

YM-B, JS-A, JA, and PL conceived the presented idea. YM-B and AR-G planned and carried out the transcriptome analysis. YM-B, JS-A, AT, JT, and FR carried out the metabolome analysis. EB and YM-B performed the DNA-protein binding assays. PL, JS-A, YM-B, and AT wrote the manuscript.

## Supplementary Material

The Supplementary Material for this article can be found online at: https://www.frontiersin.org/articles/10.3389/fmicb.2019.00580/full#supplementary-material

## Conflict of Interest Statement

The authors declare that the research was conducted in the absence of any commercial or financial relationships that could be construed as a potential conflict of interest.

## Footnotes

1 http://gnps.ucsd.edu

2 https://gnps.ucsd.edu/ProteoSAFe/status.jsp?task=cd3b6dd0aee842c1bfff1a1c48db3649

3 https://gnps.ucsd.edu/ProteoSAFe/status.jsp?task=cd3b6dd0aee842c1bfff1a1c48db3649

## References

[B1] Aidoo K. A. Wong A. Alexander D. C. Rittammer R. A. Jensen S. E. (1994). Cloning, sequencing and disruption of a gene from *Streptomyces clavuligerus* involved in clavulanic acid biosynthesis. *Gene* 147 41–46. 10.1016/0378-1119(94)90036-1 8088547

[B2] Aigle B. Wietzorrek A. Takano E. Bibb M. J. (2000). A single amino acid substitution in region 1.2 of the principal sigma factor of *Streptomyces coelicolor* A3(2) results in pleiotropic loss of antibiotic production. *Mol. Microbiol.* 37 995–1004. 10.1046/j.1365-2958.2000.02022.x 10972819

[B3] Álvarez-Álvarez R. Botas A. Albillos S. M. Rumbero A. Martín J. F. Liras P. (2015). Molecular genetics of naringenin biosynthesis, a typical plant secondary metabolite produced by *Streptomyces clavuligerus*. *Microb. Cell Fact.* 14:178. 10.1186/s12934-015-0373-7 26553209 PMC4640377

[B4] Álvarez-Álvarez R. Rodríguez-García A. Martínez-Burgo Y. Robles-Reglero V. Santamarta I. Pérez-Redondo R. (2014). A 1.8-Mb-reduced *Streptomyces clavuligerus* genome: relevance for secondary metabolism and differentiation. *Appl. Microbiol. Biotechnol.* 98 2183–2195. 10.1007/s00253-013-5382-z 24305736

[B5] Antón N. Santos-Aberturas J. Mendes M. V. Guerra S. M. Martín J. F. Aparicio J. F. (2007). PimM, a PAS domain positive regulator of pimaricin biosynthesis in *Streptomyces natalensis*. *Microbiology* 153(Pt 9), 3174–3183. 10.1099/mic.0.2007/009126-0 17768260

[B6] Baggaley K. H. Brown A. G. Schofield C. J. (1997). Chemistry and biosynthesis of clavulanic acid and other clavams. *Natl. Prod. Rep.* 140 309–333. 10.1039/NP9971400309 9281835

[B7] Barona-Gómez F. Wong U. Giannakopulos A. E. Derrick P. J. Challis G. L. (2004). Identification of a cluster of genes that directs desferrioxamine biosynthesis in *Streptomyces coelicolor* M145. *J. Am. Chem. Soc.* 126 16282–16283. 10.1021/ja045774k 15600304

[B8] Barreales E. G. Vicente C. M. de Pedro A. Santos-Aberturas J. Aparicio J. F. (2018). Promoter engineering reveals the importance of heptameric direct repeats for DNA binding by *Streptomyces* antibiotic regulatory protein-large ATP-binding regulator of the LuxR family (SARP-LAL) regulators in *Streptomyces natalensis*. *Appl. Environ. Microbiol.* 84:e246-18. 10.1128/AEM.00246-18 29500267 PMC5930380

[B9] Carmody M. Byrne B. Murphy B. Breen C. Lynch S. Flood E. (2004). Analysis and manipulation of amphotericin biosynthetic genes by means of modified phage KC515 transduction techniques. *Gene* 343 107–115. 10.1016/j.gene.2004.08.006 15563836

[B10] Chen S. Huang X. Zhou X. Bai L. He J. Jeong K. J. (2003). Organizational and mutational analysis of a complete FR-008/candicidin gene cluster encoding a structurally related polyene complex. *Chem. Biol.* 10 1065–1076. 10.1016/j.chembiol.2003.10.007 14652074

[B11] Chen W. Qu D. Zhai L. Tao M. Wang Y. Lin S. (2010). Characterization of the tunicamycin gene cluster unveiling unique steps involved in its biosynthesis. *Protein Cell* 1 1093–1105. 10.1007/s13238-010-0127-6 21153459 PMC4875072

[B12] Cheng Z. Bown L. Tahlan K. Bignell D. R. (2015). Regulation of coronafacoyl phytotoxin production by the PAS-LuxR family regulator CfaR in the common scab pathogen *Streptomyces scabies*. *PLoS One* 10:e0122450. 10.1371/journal.pone.0122450 25826255 PMC4380410

[B13] Dong Y. Y. Wang H. Pike A. C. W. Cochrane S. A. Hamedzadeh S. Wyszyñski F. J. (2018). Structures of DPAGT1 Explain Glycosylation Disease Mechanisms and Advance TB Antibiotic Design. *Cell* 175 1045–1058.e16. 10.1016/j.cell.2018.10.037 30388443 PMC6218659

[B14] Enguita F. J. Liras P. Leitao A. L. Martín J. F. (1996). Interaction of the two proteins of the methoxylation system involved in cephamycin C biosynthesis. *J. Biol. Chem.* 271 33225–33230. 10.1074/jbc.271.52.33225 8969179

[B15] Higgens C. E. Hamill R. L. Sands T. H. Hoehn M. M. Davis N. E. (1974). The occurrence of deacetoxycephalosporin C in fungi and streptomycetes. *J. Antibiot.* 27 298–300. 10.7164/antibiotics.27.298 4859396

[B16] Hobbs G. Frazer C. M. Gardner D. C. J. Cullum J. A. Oliver S. G. (1989). Dispersed growth of *Streptomyces* in liquid culture. *Appl. Microbiol. Biotechnol.* 31 272–277. 10.1007/BF00258408

[B17] Ikeda H. Ishikawa J. Hanamoto A. Shinose M. Kikuchi H. Shiba T. (2003). Complete genome sequence and comparative analysis of the industrial microorganism *Streptomyces avermitilis*. *Nat. Biotechnol.* 21 526–531. 10.1038/nbt820 12692562

[B18] Kamogashira T. Takegata S. Sugiura K. (1988). Isolation of tunicamycin produced by *Bacillus cereus* K-279. *Agric. Biol. Chem.* 52 859–861. 10.1080/00021369.1988.10868752

[B19] Kenig M. Reading C. (1979). Holomycin and an antibiotic (MM19290) related to tunicamycin, metabolites of *Streptomyces clavuligerus*. *J. Antibiot.* 36 549–554. 10.7164/antibiotics.32.549 468729

[B20] Kieser T. Bibb M. J. Buttner M. J. Chater K. F. Hopwood D. A. (2000). *Practical Streptomyces Genetics.* Norwich: John Innes Foundation.

[B21] Lee C. Kim J. Shin S. G. Hwang S. (2006). Absolute and relative qPCR quantification of plasmid copy number in *Escherichia coli*. *J. Biotechnol.* 123 273–280. 10.1016/j.jbiotec.2005.11.014 16388869

[B22] Li L. Zhao Y. Ruan L. Yang S. Ge M. Jiang W. (2015). A stepwise increase in pristinamycin II biosynthesis by *Streptomyces pristinaespiralis* through combinatorial metabolic engineering. *Metab. Eng.* 29 12–25. 10.1016/j.ymben.2015.02.001 25708513

[B23] Liras P. (1999). Biosynthesis and Molecular Genetics of Cephamycins. Cephamycins produced by actinomycetes. *Antonie Van Leeuwenhoek.* 75 109–124. 10.1023/A:1001804925843 10422584

[B24] Liras P. (2014). Holomycin, a dithiolopyrrolone compound produced by *Streptomyces clavuligerus*. *Appl. Microbiol. Biotechnol.* 98 1023–1030. 10.1007/s00253-013-5410-z 24323287

[B25] Liras P. Gomez-Escribano J. P. Santamarta I. (2008). Regulatory mechanisms controlling antibiotic production in *Streptomyces clavuligerus*. *J. Ind. Microbiol. Biotechnol.* 35 667–676. 10.1007/s10295-008-0351-8 18446393

[B26] Livak K. J. Schmittgen T. D. (2001). Analysis of relative gene expression data using real time quantitative PCR and the 2-(Ct Method. *Methods* 25 402–408. 10.1006/meth.2001.1262 11846609

[B27] López-García M. T. Santamarta I. Liras P. (2010). Morphological differentiation and clavulanic acid formation are affected in a *Streptomyces clavuligerus adpA*-deleted mutant. *Microbiology* 156 2354–2365. 10.1099/mic.0.035956-0 20447998

[B28] Lorenzana L. M. Pérez-Redondo R. Santamarta I. Martín J. F. Liras P. (2004). Two oligopeptide-permease-encoding genes in the clavulanic acid cluster of *Streptomyces clavuligerus* are essential for production of the beta-lactamase inhibitor. *J. Bacteriol.* 186 3431–3438. 10.1128/JB.186.11.3431-3438.2004 15150229 PMC415745

[B29] Lu C. Zhang X. Jiang M. Bai L. (2016). Enhanced salinomycin production by adjusting the supply of polyketide extender units in *Streptomyces albus*. *Metab. Eng.* 35 129–137. 10.1016/j.ymben.2016.02.012 26969249

[B30] Martín J. F. Liras P. (2015). “Novel antimicrobial and other bioactive metabolites obtained from silent gene clusters,” in *Antibiotics. Current Innovations and Future Trends*, eds By Sanchez S. Demain A. L. (Poole: Caister Academic Press).

[B31] Martínez-Burgo Y. Álvarez-Álvarez R. Rodríguez-García A. Liras P. (2015). The pathway-specific regulator ClaR of *Streptomyces clavuligerus* has a global effect on the expression of genes for secondary metabolism and differentiation. *Appl. Environ. Microbiol.* 81 6637–6648. 10.1128/AEM.00916-15 26187955 PMC4561703

[B32] McLean T. C. Hoskisson P. A. Seipke R. F. (2016). Coordinate regulation of antimycin and candicidin biosynthesis. *mSphere* 1 e305-16. 10.1128/mSphere.00305-16 27981234 PMC5143413

[B33] Medema M. H. Alam M. T. Heijne W. H. van den Berg M. A. Müller U. Trefzer A. (2011). Genome-wide gene expression changes in an industrial clavulanic acid overproduction strain of *Streptomyces clavuligerus*. *Microb. Biotechnol.* 4 300–305. 10.1111/j.1751-7915.2010.00226.x 21342474 PMC3818869

[B34] Medema M. H. Trefzer A. Kovalchuk A. van den Berg M. Müller U. Heijne W. (2010). The sequence of a 1.8-Mb bacterial linear plasmid reveals a rich evolutionary reservoir of secondary metabolic pathways. *Genome Biol Evol.* 2 212–224. 10.1093/gbe/evq013 20624727 PMC2997539

[B35] Medina-Rivera A. Defrance M. Sand O. Herrmann C. Castro-Mondragon J. A. Delerce J. (2015). RSAT 2015: regulatory sequence analysis tools. *Nucleic Acids Res.* 43 W50–W56. 10.1093/nar/gkv362 25904632 PMC4489296

[B36] Mehra S. Lian W. Jayapal K. P. Charaniya S. P. Sherman D. H. Hu W. S. (2006). A framework to analyze multiple time series data: a case study with *Streptomyces coelicolor*. *J. Ind. Microbiol. Biotechnol.* 33 159–172. 10.1007/s10295-005-0034-7 16217633

[B37] Nguyen D. D. Wu C. H. Moree W. J. Lamsa A. Medema M. H. Zhao X. (2013). MS/MS networking guided analysis of molecule and gene cluster families. *Proc. Natl. Acad. Sci. U.S.A.* 110 E2611–E2620. 10.1073/pnas.1303471110 23798442 PMC3710860

[B38] Olano C. García I. González A. Rodriguez M. Rozas D. Rubio J. (2014). Activation and identification of five clusters for secondary metabolites in *Streptomyces albus* J1074. *Microb. Biotechnol.* 7 242–256. 10.1111/1751-7915.12116 24593309 PMC3992020

[B39] Omura S. Ikeda H. Ishikawa J. Hanamoto A. Takahashi C. Shinose M. (2001). Genome sequence of an industrial microorganism *Streptomyces avermitilis*: deducing the ability of producing secondary metabolites. *Proc. Natl. Acad. Sci. U.S.A.* 98 12215–12220. 10.1073/pnas.211433198 11572948 PMC59794

[B40] Ordóñez-Robles M. Rodríguez-García A. Martín J. F. (2016). Target genes of the *Streptomyces tsukubaensis* FkbN regulator include most of the tacrolimus biosynthesis genes, a phosphopantetheinyl transferase and other PKS genes. *Appl. Microbiol. Biotechnol.* 100 8091–8103. 10.1007/s00253-016-7696-0 27357227

[B41] Patterson S. I. Skene J. H. P. (1994). Novel inhibitory action of tunicamycin homologues suggests a role for dynamic protein fatty acylation in growth cone-mediated neurite extension. *J. Cell Biol.* 124 521–536. 10.1083/jcb.124.4.521 8106550 PMC2119910

[B42] Payero T. D. Vicente C. M. Rumbero Á Barreales E. G. Santos-Aberturas J. de Pedro A. (2015). Functional analysis of filipin tailoring genes from *Streptomyces filipinensis* reveals alternative routes in filipin III biosynthesis and yields bioactive derivatives. *Microb. Cell Fact.* 14:114. 10.1186/s12934-015-0307-4 26246267 PMC4527110

[B43] Pérez-Redondo R. Rodríguez-García A. Martín J. F. Liras P. (1998). The *cla*R gene of *Streptomyces clavuligerus*, encoding a LysR-type regulatory protein controlling clavulanic acid biosynthesis, is linked to the clavulanate-9-aldehyde reductase (*car*) gene. *Gene* 211 311–321. 10.1016/S0378-1119(98)00106-1 9602162

[B44] Pospiech A. Neumann B. (1995). A versatile quick-prep of genomic DNA from gram-positive bacteria. *Trends Genet.* 11 217–218. 10.1016/S0168-9525(00)89052-6 7638902

[B45] Price N. B. J. Tsvetanova B. (2007). Biosynthesis of the tunicamycins: a review. *J. Antibiot.* 60 485–491. 10.1038/ja.2007.62 17827659

[B46] Rodríguez-García A. Barreiro C. Santos-Beneit F. Sola-Landa A. Martín J. F. (2007). Genome-wide transcriptomic and proteomic analysis of the primary response to phosphate limitation in *Streptomyces coelicolor* M145 and in a Δ*phoP* mutant. *Proteomics* 7 2410–2429. 10.1002/pmic.200600883 17623301

[B47] Sambrook J. Russell D. (2001). *Molecular Cloning: A Laboratory Manual, Third Edn.* Cold Spring Harbor, NY: Cold Spring Harbor Lab. Press.

[B48] Sánchez L. Braña A. F. (1996). Cell density influences antibiotic biosynthesis in *Streptomyces clavuligerus*. *Microbiology* 142 1209–1220. 10.1099/13500872-142-5-1209 8704961

[B49] Santos-Aberturas J. Payero T. D. Vicente C. M. Guerra S. M. Cañibano C. Martín J. F. (2011a). Functional conservation of PAS-LuxR transcriptional regulators in polyene macrolide biosynthesis. *Metab. Eng.* 13 756–757. 10.1016/j.ymben.2011.09.011 22001323

[B50] Santos-Aberturas J. Vicente C. M. Guerra S. M. Payero T. D. Martín J. F. Aparicio J. F. (2011b). Molecular control of polyene macrolide biosynthesis: direct binding of the regulator PimM to eight promoters of pimaricin genes and identification of binding boxes. *J. Biol. Chem.* 286 9150–9161. 10.1074/jbc.M110.182428 21187288 PMC3059063

[B51] Santos-Aberturas J. Vicente C. M. Payero T. D. Martín-Sánchez L. Cañibano C. Martín J. F. (2012). Hierarchical control on polyene macrolide biosynthesis: PimR modulates pimaricin production via the PAS-LuxR transcriptional activator PimM. *PLoS One* 7:e38536. 10.1371/journal.pone.0038536 22693644 PMC3367932

[B52] Sartor M. Schwanekamp J. Halbleib D. Mohamed I. Karyala S. Medvedovic M. (2004). Microarray results improve significantly as hybridization approaches equilibrium. *Biotechniques* 36 790–796. 10.2144/04365ST02 15152598

[B53] Schmittgen T. D. Zakrajsek B. A. (2000). Effect of experimental treatment on housekeeping gene expression: validation by real-time, quantitative RT-PCR. *J. Biochem. Biophys. Methods* 46 69–81. 10.1016/S0165-022X(00)00129-9 11086195

[B54] Schneider T. D. (1997). Information content of individual genetic sequences. *J. Theor. Biol.* 189 427–441. 10.1006/jtbi.1997.0540 9446751

[B55] Shao L. Huang J. J. Yu Y. Li M. X. Pu T. Kan S. D. (2014). Improvement of 7α-methoxycephalosporins production by overexpression of *cmcJ* and *cmcI* controlled by promoter *ermEp*^∗^ in *Streptomyces clavuligerus*. *J. Appl. Microbiol.* 117 1645–1654. 10.1111/jam.12640 25179172

[B56] Shirling S. B. Gottlieb D. (1966). Methods for characterization of Streptomyces species. *Int. J. Syst. Bacteriol.* 16 313–340. 10.1099/00207713-16-3-313

[B57] Takatsuki A. Arima K. Tamura G. (1971). Tunicamycin, a new antibiotic. I. Isolation and characterization of tunicamycin. *J. Antibiot.* 24 215–223. 10.7164/antibiotics.24.215 5572750

[B58] Thompson C. J. Ward J. M. Hopwood D. A. (1980). DNA cloning in *Streptomyces*: resistance genes from antibiotic-producing species. *Nature* 286 525–527. 10.1038/286525a0 6250070

[B59] Thompson C. J. Ward J. M. Hopwood D. A. (1982). Cloning of antibiotic resistance and nutritional genes in streptomycetes. *J. Bacteriol.* 151 668–677. 6284706 10.1128/jb.151.2.668-677.1982 PMC220307

[B60] Tsvetanova B. C. Price N. P. J. (2001). Liquid Chromatography–electrospray mass spectrometry of tunicamycin-type antibiotics. *Anal. Biochem.* 289 147–156. 10.1006/abio.2000.4952 11161308

[B61] Vicente C. M. Payero T. D. Santos-Aberturas J. Barreales E. G. de Pedro A. Aparicio J. F. (2015). Pathway-specific regulation revisited: cross-regulation of multiple disparate gene clusters by PAS-LuxR transcriptional regulators. *Appl. Microbiol. Biotechnol.* 99 5123–5135. 10.1007/s00253-015-6472-x 25715784

[B62] Vicente C. M. Santos-Aberturas J. Payero T. D. Barreales E. G. de Pedro A. Aparicio J. F. (2014). PAS-LuxR transcriptional control of filipin biosynthesis in S. *avermitilis*. *Appl. Microbiol. Biotechnol.* 98 9311–9324. 10.1007/s00253-014-5998-7 25104037

[B63] Wang M. Carver J. J. Phelan V. V. Sanchez L. M. Garg N. Peng Y. (2016). Sharing and community curation of mass spectrometry data with global natural products social molecular networking. *Nat. Biotechnol.* 34 828–837. 10.1038/nbt.3597 27504778 PMC5321674

[B64] Wang Y. Tao Z. Zheng H. Zhang F. Long Q. Deng Z. (2016). Iteratively improving natamycin production in *Streptomyces gilvosporeus* by a large operon-reporter based strategy. *Metab. Eng.* 38 418–426. 10.1016/j.ymben.2016.10.005 27746324

[B65] Widdick D. Royer S. F. Wang H. Vior N. M. Gomez-Escribano J. P. Davis B. G. (2018). Analysis of the tunicamycin biosynthetic gene cluster of *Streptomyces chartreusis* reveals new insights into tunicamycin production and immunity. *Antimicrob. Ageing Chemother.* 62:e130-18. 10.1128/AAC.00130-18 29844049 PMC6105854

[B66] Wilkinson C. J. Hughes-Thomas Z. A. Martin C. J. Bohm I. Mironenko T. Deacon M. (2002). Increasing the efficiency of heterologous promoters in actinomycetes. *J. Mol. Microbiol. Biotechnol.* 4 417–426. 12125822

[B67] Wyszynski F. J. Hesketh A. R. Bibb M. J. Davis B. G. (2010). Dissecting tunicamycin biosynthesis by genome mining: cloning and heterologous expression of a minimal gene cluster. *Chem. Sci.* 1 581–589. 10.1039/C0SC00325E

[B68] Yagüe P. Rodríguez-García A. López-García M. T. Rioseras B. Martín J. F. Sánchez J. (2014). Transcriptomic analysis of liquid non-sporulating *Streptomyces coelicolor* cultures demonstrates the existence of a complex differentiation comparable to that occurring in solid sporulating cultures. *PLoS One* 9:e86296. 10.1371/journal.pone.0086296 24466012 PMC3897704

[B69] Zabala D. Braña A. F. Flórez A. B. Salas J. A. Méndez C. (2013). Engineering precursor metabolite pools for increasing production of antitumor mithramycins in *Streptomyces argillaceus*. *Metab. Eng.* 20 187–197. 10.1016/j.ymben.2013.10.002 24148183

