# Supplementary material for: Activation of Secondary Metabolite Gene Clusters in Streptomyces clavuligerus by the PimM Regulator of Streptomyces natalensis
Source: Front Microbiol. 2019 Mar 26;10:580. doi: 10.3389/fmicb.2019.00580 (PMC6448028; doi:10.3389/fmicb.2019.00580)
Supplement: Supplementary file 6 [file Image_1.pdf]

SUPPLEMENTARY MATERIAL

Activation of secondary metabolite gene clusters in *Streptomyces clavuligerus* by the PimM regulator of *Streptomyces natalensis*

Yolanda Martínez-Burgo, Javier Santos-Aberturas, Antonio Rodríguez-García, Eva G. Barreales, José Rubén Tormo, Andrew W. Truman, Fernando Reyes, Jesús F. Aparicio, and Paloma Liras

*** Correspondence:** Corresponding Author: paloma.liras@unileon.es

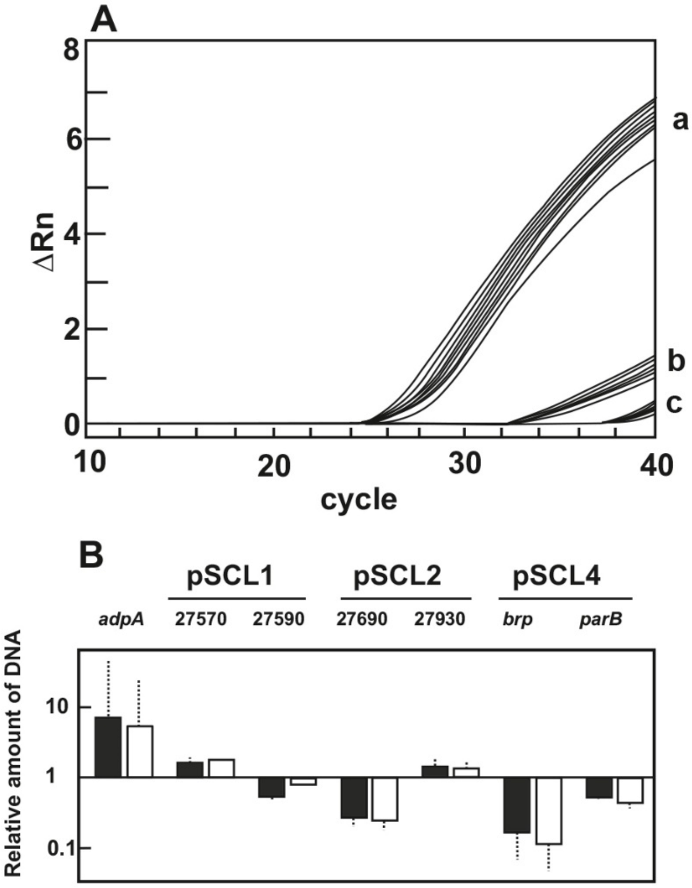

Fig. S1. Martínez-Burgo Y et al.

Figure S1. Characterization of *S. clavuligerus*::*pimM*. (A) Analysis of *pimM* expression by RT-qPCR. Normalized reporter value (ΔRn) vs number of cycles (Ct) curves of a) *rpoD* in *S. clavuligerus*::*pimM* and *S. clavuligerus*::pIB139; b) *pimM* in *S. clavuligerus*::*pimM*; and c) *pimM* in *S. clavuligerus*::pIB139. Curves correspond to the two biological and three technical replicates. (B) Relative amount of DNA of pSCL1, pSCL2, and pSCL4 in *S. clavuligerus*::*pimM* and *S. clavuligerus*::pIB139 by comparison with the wild-type *S. clavuligerus* ATCC 27064. For each plasmid the relative amount of DNA of the genes indicated on top of the graph was analyzed by qPCR. S. *clavuligerus*::pIB139 is represented by black columns and *S. clavuligerus*::*pimM* by white ones. Vertical lines indicate standard deviations.

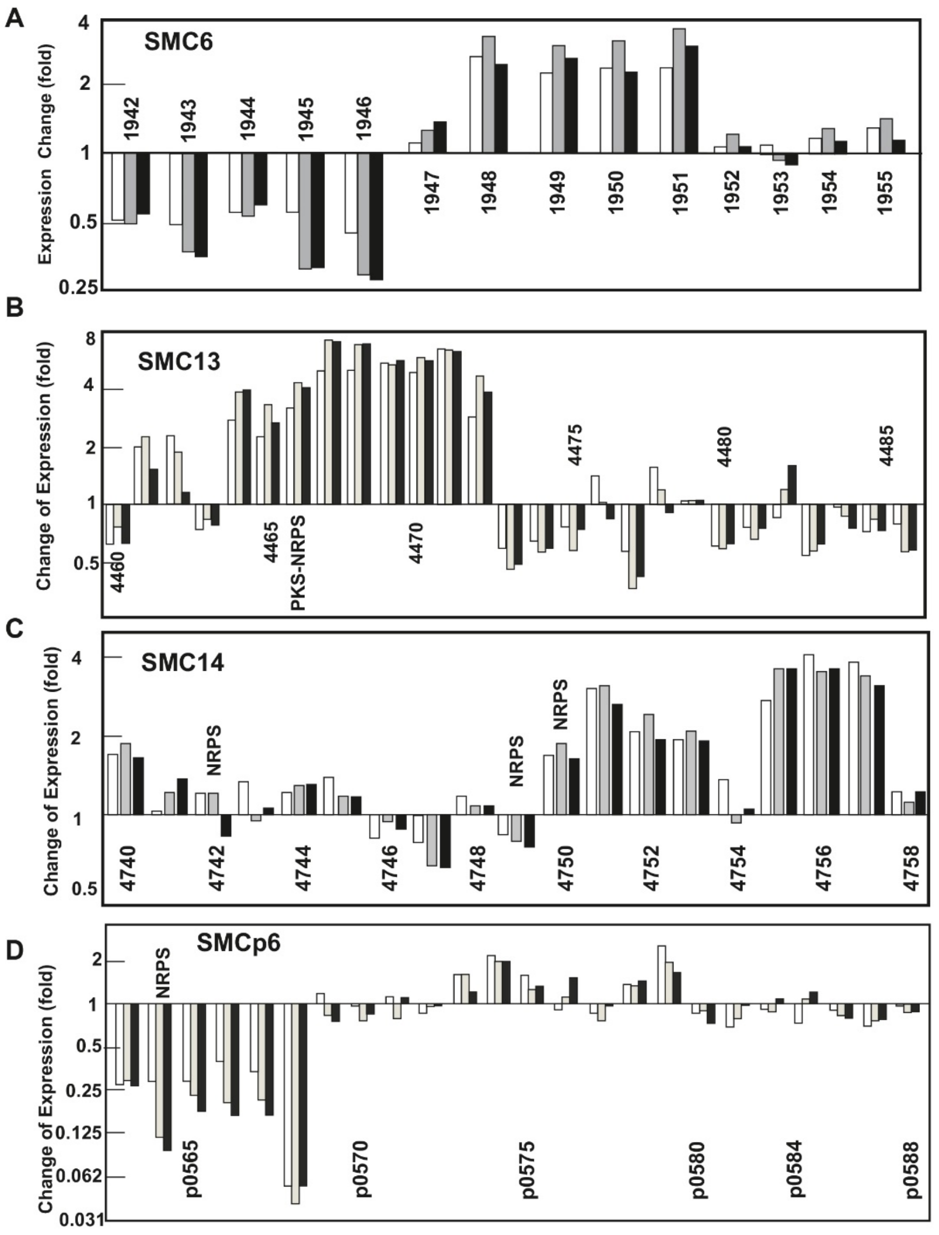

Fig. S2. Martínez-Burgo Y et al.

Figure S2. Effect of PimM on the transcription level of SMC6, SMC13, SMC14 and SMCp6. Change in the expression level of (A) SMC6, (B) SMC13, (C) SMC14 and (D) SMCp6 in *S. clavuligerus*::*pimM*. In the panels the columns represent the average of the expression fold-change at 84 h (white columns), 90 h (gray columns), and 96 h (black columns). Numbering of the SCLAV genes is indicated. Expression level values of *S. clavuligerus*::*pimM* are compared to those of the control strain, *S. clavuligerus*::pIB139, taken as 1. Genes encoding hybrid polyketide synthases-nonribosomal peptide synthetases and non-ribosomal peptide synthetases are indicated by the letters PKS-NRPS and NRPS, respectively.

Figure S3. Validation by RT-qPCR of the transcriptomic results

A)

Gene	Product	M_c	$\log_2 2^{-\Delta\Delta Ct}$
SCLAV_0142	Serine/threonine protein kinase	-3.00	-3.38
SCLAV_0833	Phenylacetaldehyde dehydrogenase	-3.63	-4.62
SCLAV_0835	Transcriptional regulator	-2.43	-1.45
SCLAV_1951	Nocardamine synthetase (Nocardamine)	1.78	2.18
SCLAV_4197	Carboxyethylarginine synthase (Clavulanic acid)	1.70	2.31
SCLAV_4207	Methyl transferase (Cephamycin C)	2.20	2.79
SCLAV_4466	Hybrid NRPS / PKS (SMC13)	2.11	1.59
SCLAV_5308	Transcription factor jumonji jmjC domain protein	-2.41	-3.36
SCLAV_p0511	Type II PKS (SMCp5)	1.64	1.95
SCLAV_p0520	SGL domain-containing protein (SMCp5)	1.86	2.54
SCLAV_p0566	3-oxoacyl-acp synthase (SMCp6)	-2.30	-3.06
SCLAV_p0568	IclR family transcriptional regulator (SMCp6)	-4.64	-4.53

B)

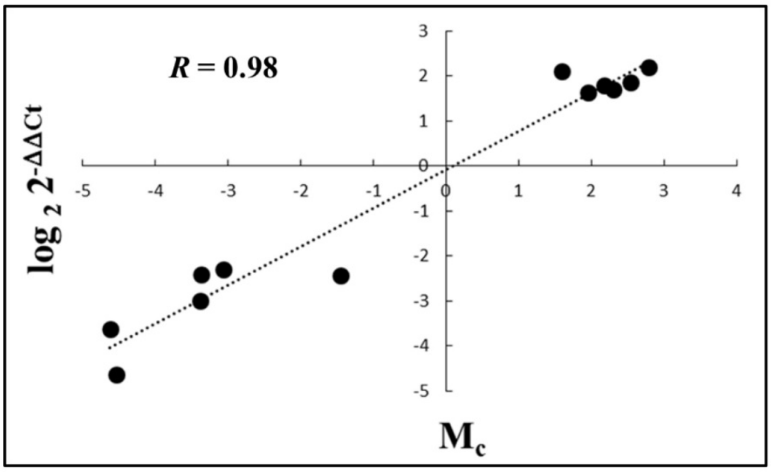

Fig. S3. Martínez-Burgo Y et al.

Figure S3. Validation of the transcriptomic data. (A) Comparison of the data obtained for each gene analysed in the microarray experiment (M_c) and by RT-qPCR ($\log_2 2^{-\Delta\Delta Ct}$) in *S. clavuligerus*::*pimM vs S. clavuligerus*::pIB139. For those genes that are in secondary metabolite gene clusters, the gene cluster is indicated. (B) Graphical representation of the correlation between the results shown in panel A.

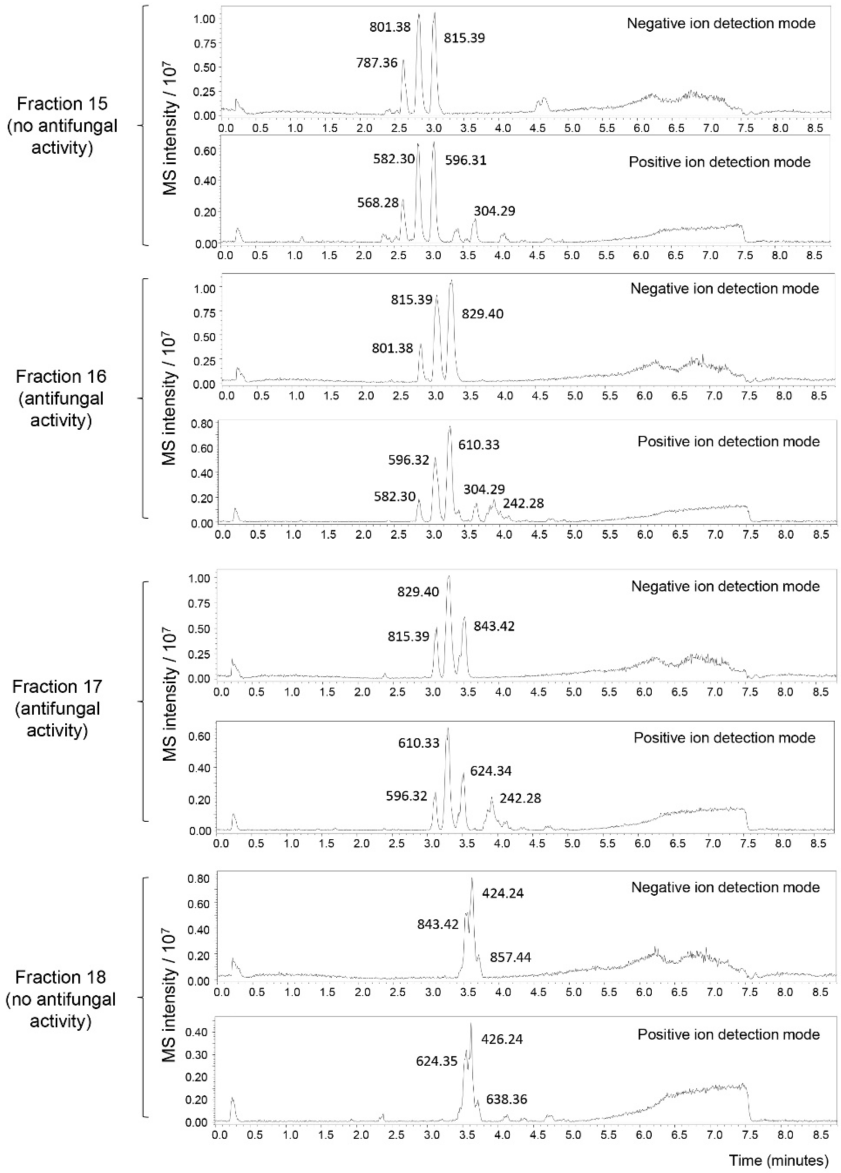

Fig. S4. Martínez-Burgo Y et al.

Figure S4. LC-MS analysis of the antifungal HPLC fractions. LC-MS analysis of fractions 16 and 17 (exhibiting antifungal activity in the bioassays) alongside fractions 15 and 18 (with no antifungal activity), both in positive and negative ion detection modes.

A

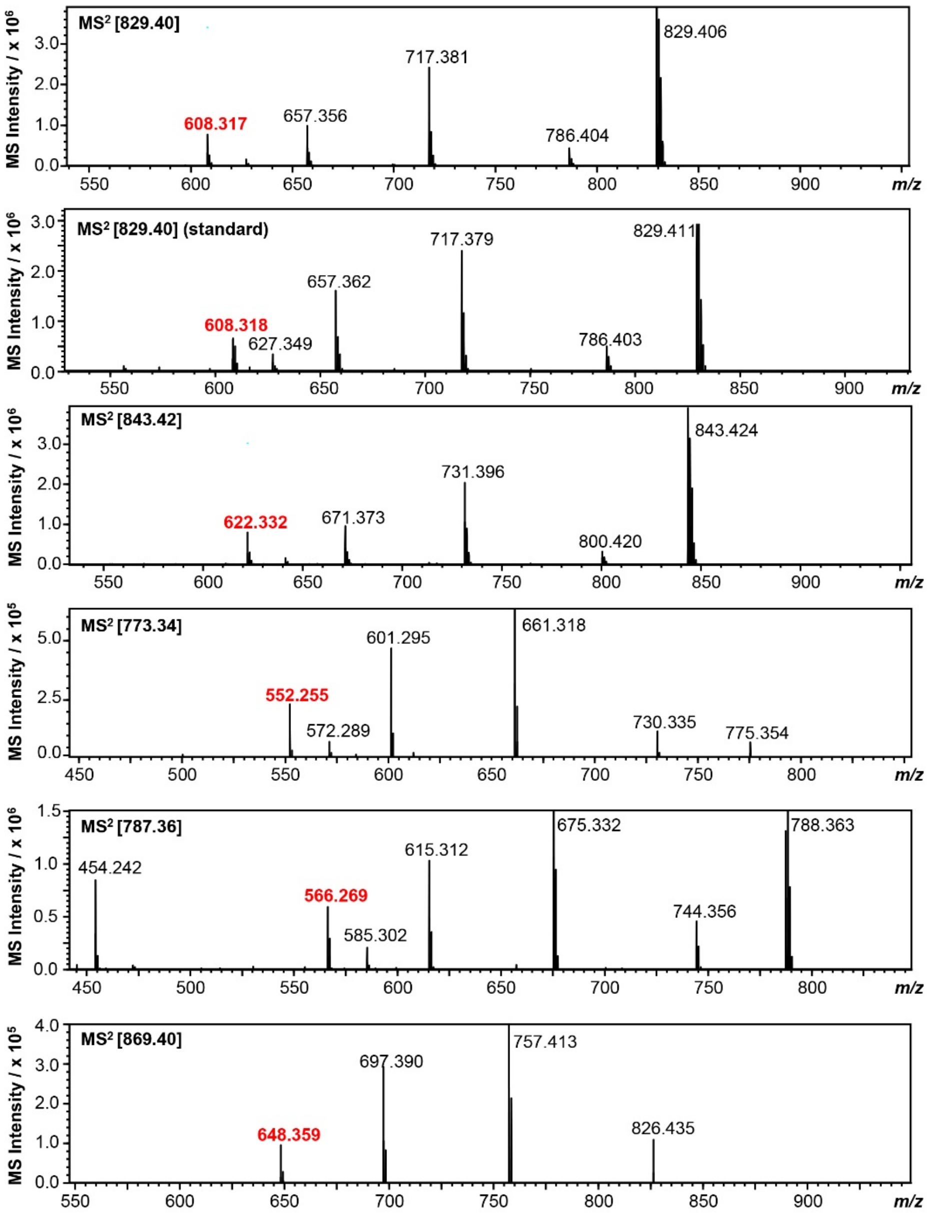

B

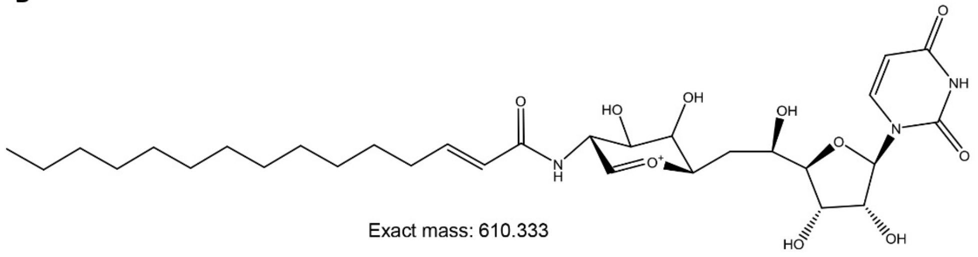

Tunicamycin IV (Pseudo-MS² fragmentation in positive ion detection mode)

Fig. S5. Martínez-Burgo Y et al.

Figure S5. MS^2 fragmentation assisted identification of known and new tunicamycins. (A) The MS^2 fragmentation in negative ion detection mode is shown for two known tunicamycins (*m/z* 829.41 and 843.43), a tunicamycin standard (*m/z* 829.41), and three previously undescribed tunicamycin congeners (*m/z* 773.34, 787.36 and 869.44). The similarity of the MS^2 fragmentation patterns can be observed. A characteristic fragment with a loss of 221 Da with respect to the parent ion (due to the loss of the N-acetylglucosamine moiety from the parent tunicamycin) is highlighted in red. (B) In positive ion mode, source fragmentation leads to tunicamycin-derived ions that result from a characteristic loss of 221 Da, which corresponds to the N-acetylglucosamine moiety. The tunicamycin IV-derived fragment is shown.

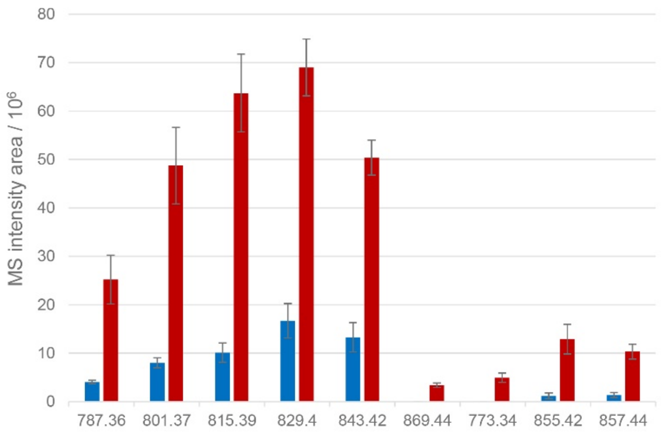

Fig. S6. Martínez-Burgo Y et al.

Figure S6. LC-MS quantification of tunicamycins. The bars represent the total ion count for each tunicamycin congener in positive ion detection mode for *S. clavuligerus*::pIB139 (blue) and *S. clavuligerus*::*pimM* (red). Error bars correspond to the standard deviation calculated from three biological replicates.
